# Empagliflozin inhibits angiotensin II-induced hypertrophy in H9c2 cardiomyoblasts through inhibition of NHE1 expression

**DOI:** 10.1007/s11010-022-04411-6

**Published:** 2022-03-25

**Authors:** Nabeel Abdulrahman, Meram Ibrahim, Jensa Mariam Joseph, Hanan Mahmoud Elkoubatry, Al-Anood Al-Shamasi, Menatallah Rayan, Alain Pierre Gadeau, Rashid Ahmed, Hussein Eldassouki, Anwarul Hasan, Fatima Mraiche

**Affiliations:** 1 College of Pharmacy, QU Health, Qatar University, P.O. Box 2713, Doha, Qatar; 2 Biomedical and Pharmaceutical Research Unit, Qatar University, Doha, Qatar; 3 Translational Research Institute, Academic Health System, Hamad Medical Corporation, Doha, Qatar; 4 University of Bordeaux, UMR1034 Pessac, France; 5 Department of Mechanical and Industrial Engineering, College of Engineering, Qatar University, 2713 Doha, Qatar; 6 College of Kinesiology, University of Saskatchewan, Saskatoon, SK Canada

**Keywords:** Empagliflozin, SGLT-1/2, Cardiomyocyte hypertrophy, NHE1, Angiotensin II, H9c2 cardiomyoblasts

## Abstract

Diabetes mellitus (DM)-induced cardiac morbidities have been the leading cause of death among diabetic patients. Recently, sodium-glucose cotransporter-2 (SGLT-2) inhibitors including empagliflozin (EMPA), which have been approved for the treatment of DM, have gained attention for their cardioprotective effect. The mechanism by which SGLT-2 inhibitors exert their cardioprotective effect remains unclear. Recent studies have suggested that EMPA exerts its cardioprotective effect by inhibiting the Na^+^/H^+^ exchanger (NHE), a group of membrane proteins that regulate intracellular pH and cell volume. Increased activity and expression of NHE isoform 1 (NHE1), the predominant isoform expressed in the heart, leads to cardiac hypertrophy. p90 ribosomal s6 kinase (p90 RSK) has been demonstrated to stimulate NHE1 activity. In our study, H9c2 cardiomyoblasts were treated with angiotensin II (ANG) to activate NHE1 and generate a hypertrophic model. We aimed to understand whether EMPA reverses the ANG-induced hypertrophic response and to elucidate the molecular pathway contributing to the cardioprotective effect of EMPA. Our study demonstrated that ANG-induced hypertrophy of H9c2 cardiomyoblasts is accompanied with increased SGLT-1 and NHE1 protein expression, an effect which is prevented in the presence of EMPA. EMPA reduces ANG-induced hypertrophy through the inhibition of SGLT-1 and NHE1 expression.

## Introduction

Diabetes mellitus (DM) is a group of metabolic disorders manifested by chronic hyperglycemia which occurs as a result of a defect in insulin secretion, insulin action or both [1]. DM is classified into type 1, which accounts for 5–10% of all the cases diagnosed with DM, and type 2 (T2DM), which accounts for 90–95% of all cases [1, 2]. The prevalence of DM is increasing in such a way that a total of 592 million people would suffer from DM by the year 2035 [3]. DM causes cardiomyopathy which is manifested by an increased ventricular hypertrophy and diastolic dysfunction and may lead to heart failure [4]. Consequently, the associated cardiac morbidities are a leading cause of death among diabetic patients.

Sodium-glucose cotransporter-2 (SGLT-2) inhibitors, a novel class of anti-diabetic drugs which inhibit glucose reabsorption from the proximal convoluted tubule of the kidney, have gained attention for their cardioprotective effect. It was reported that SGLT-2 inhibitors, empagliflozin (EMPA) and dapagliflozin (DAPA), reduced the lipotoxic damage in stearate treated myeloid angiogenic cells, and reduced ADP stimulated platelet activation, resulting in cardiovascular protection through plaque stabilization and thrombosis inhibition [5]. Randomized controlled trials have resulted in a significant reduction in cardiovascular events and mortality following treatment with EMPA and another SGLT-2 inhibitor, canagliflozin; as reported in Empagliflozin Cardiovascular Outcome Event Trial in Type 2 Diabetes Mellitus patients (EMPA-REG OUTCOME) and The CANagliflozin cardioVascular Assessment Study (CANVAS) [6]. DAPA has been used in a clinical trial with patients with DM who were at risk with atherosclerotic cardiovascular disease, and resulted in a lower rate of cardiovascular death or hospitalization for heart failure [7]. The mechanism by which SGLT-2 inhibitors exert their cardioprotective effect remains unclear.

NHEs are a group of membrane proteins that mediate the exchange of one intracellular H^+^ for one extracellular Na^+^ thereby regulating intracellular pH and cell volume. There are ten isoforms of NHEs, out of which NHE1 is the predominant plasma membrane isoform expressed in the myocardium [8]. Increased activity of NHE1 elicits gene expression that leads to cardiac hypertrophy [8]. Furthermore, a study has shown that infection of H9c2 cardiomyoblasts with the active form of NHE1 resulted in cardiomyocyte hypertrophy [9]. Specific silencing of myocardial NHE with short hairpin RNA reduced cardiac hypertrophy in rats [10]. Unfortunately, in clinical trials, NHE1 inhibitors resulted in serious cerebrovascular side effects in patients with coronary artery disease [11]. Identifying whether EMPA mediates its cardioprotective effect through inhibition of NHE1 and exploring the signaling pathways that may lead to the indirect inhibition of NHE1 would be highly desirable.

Previous studies have identified that the cardioprotective effects of EMPA are exerted by the inhibition of NHE [12, 13]. Conversely, a very recent study has also reported that EMPA does not inhibit the activity of NHE1, nor has any effect on intracellular sodium over a wide range of concentrations [14]. In this study, the expression of NHE1 protein was not measured. In our study, we aimed to further understand the role of NHE1 in mediating the cardioprotective effect of EMPA and to determine whether protein expression of NHE1 in H9c2 cardiomyoblasts can be prevented in the presence of EMPA. EMPA directly inhibited cardiac NHE1 flux and reduced cytoplasmic Na^+^ concentration possibly through direct binding to the Na^+^ binding site [15]. EMPA has also restored the antiapoptotic activity of XIAP and BIRC5 and demonstrated a cardioprotective effect independent of the presence of diabetes, mainly through its inhibitory effect on NHE1. This was demonstrated using artificial intelligence accompanied by in vivo validation [16].

In our study, angiotensin II (ANG) was used to treat H9c2 cardiomyoblasts to generate a hypertrophic in vitro model [17–19]. Moreover, ANG impairs insulin sensitivity and contributes to microvascular diseases in DM [13]. The objective of this study was to observe whether EMPA reverses the ANG-induced hypertrophic response and to elucidate the involvement of NHE1 protein expression in the cardioprotective effect of EMPA.

## Materials and methods

### Materials

All routine chemicals and consumables were purchased from BD Biosciences (San Jose, CA. USA), Fisher Scientific (Waltham, MA, USA), or Sigma (St. Louis, MO, USA). Primary antibodies SGLT-1 (ab14686) polyclonal, SGLT-2 (ab85626) polyclonal, NHE1 (ab126725) monoclonal, alpha tubulin (catalog no. ab4074) polyclonal were obtained from Abcam (Cambridge, MA, USA); phospho p90 RSK (catalog no. 9341) polyclonal and phospho-Akt (catalog no. 9271) polyclonal were obtained from Cell signaling (Beverly, MA, USA); and RSK2 (catalog no. 1430) was obtained from Santa Cruz Biotechnology (Dallas, TX, USA). Angiotensin II (ANG) was obtained from Sigma (St. Louis, MO, USA). SGLT-2 inhibitor, EMPA, was obtained from Cayman Chemical, Michigan, USA. EMPA solution was prepared in DMSO.

### Cell culture of H9c2 cardiomyoblasts

9c2 cardiomyoblasts, derived from embryonic BDIX rat heart tissue [20] were obtained from European Collections of Cell Cultures (ECACC). H9c2 cells showed similar properties of primary rat neonatal cardiomyocytes including hypertrophic traits when stimulated with hypertrophic agents in vitro [21, 22]. The cells were cultured in tissue culture flasks in Dulbecco’s Modified Eagle Medium (DMEM) supplemented with 10% fetal bovine serum (FBS), 1% penicillin–streptomycin, in a humidified atmosphere of 95% air–5% CO_2_ at 37 °C. Cells were treated with ANG 100 nM, empagliflozin (EMPA) 500 nM (SGLT-2 inhibitor), or a combination of ANG and EMPA for a period of 24 h. Distilled water served as a control (CTRL). Cells were grown in 35 mm dishes.

### Crystal violet staining and cell surface area measurement

Crystal violet staining of H9c2 cardiomyoblasts was performed as mentioned previously [21]. Briefly, treated cardiomyoblasts were rinsed with PBS, incubated with 4% formaldehyde for 5–10 min at room temperature, and rinsed with PBS. The cells were then incubated with cold methanol for 20 min at room temperature, rinsed with PBS, and then stained with 0.5% crystal violet in 2% ethanol. The cells were then rinsed thoroughly with PBS and observed under a light microscope. A minimum of 70 representative cells per treatment were considered, the average of which was represented as one n value. Cell surface area was calculated using AxioVision Imaging software (Carl Zeiss Microimaging).

### Western blotting

H9c2 cardiomyoblasts were lysed in ice-cold RIPA buffer along with protease inhibitors. Lysates were centrifuged at 12,000 rpm for 15 min at 4 °C. The supernatant-containing protein was collected and assayed with BioRad DC protein assay kit to determine the concentration. Equal amount of protein was separated by SDS (sodium dodecyl sulfate) polyacrylamide gels and then transferred on to a nitrocellulose membrane. The membranes were then incubated overnight at 4 °C with the following primary antibodies: anti-SGLT-1, anti-SGLT-2, anti-NHE1, anti-phospho p90 RSK, anti-RSK2, anti-phospho-Akt, or anti-Akt. Anti-alpha tubulin was used as a loading control. The membranes were then incubated in horse radish peroxidase conjugated secondary antibodies. Visualization of bands was based on enhanced chemiluminescence (ECL) reaction. Imaging was done by Fluorchem M FM0564 imager and quantification of bands was performed with Scion software.

### Statistical analysis

All values expressed were compared as a percentage of control ± SEM. A Student’s *t* test was used to calculate the differences between groups, where a *P* value ≤ 0.05 was considered a significant difference.

## Results

### ANG-induced increase in cell surface area of H9c2 cardiomyoblasts was reduced in the presence of EMPA

**Fig. 1:**
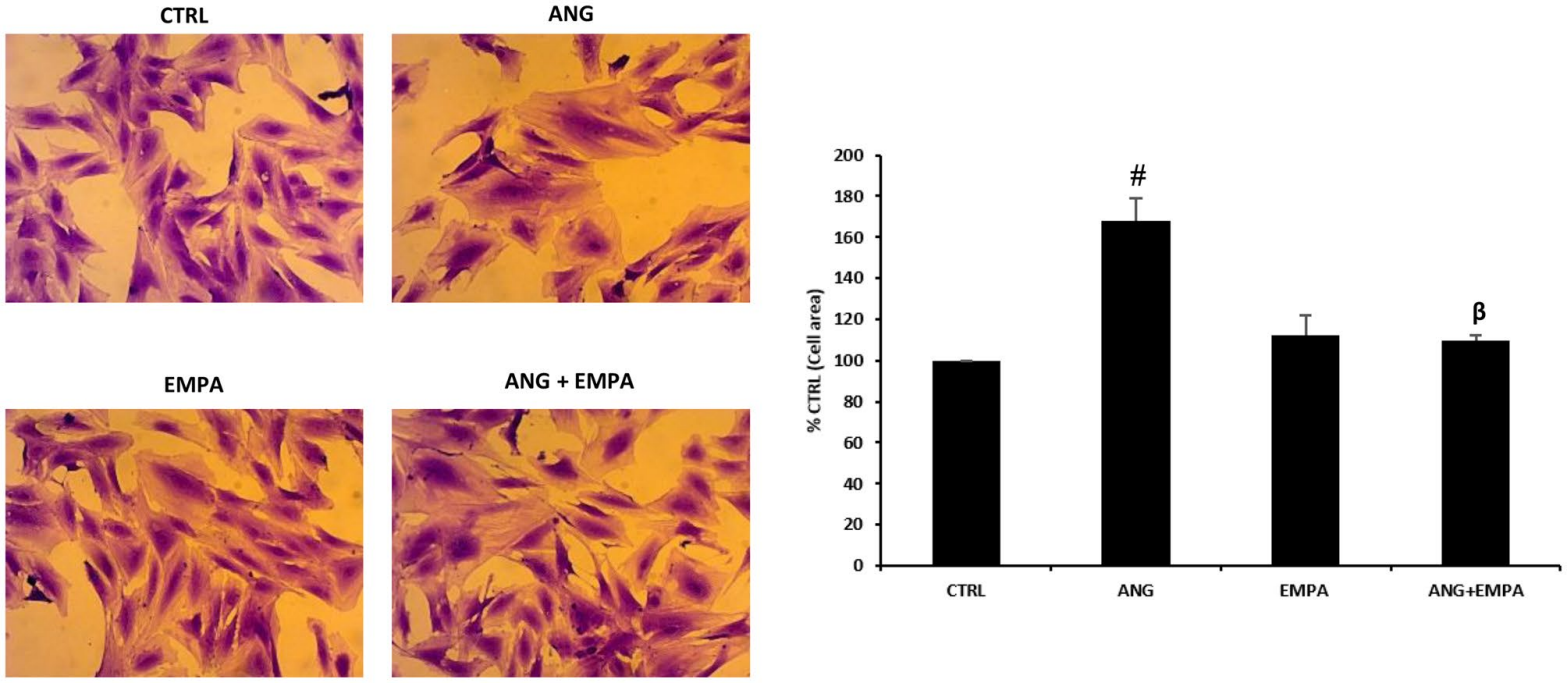
ANG-induced increase in cell surface area of H9c2 cardiomyoblasts was reduced in the presence of EMPA. *Left*: Representative images of H9c2 cardiomyoblasts stained with crystal violet. *Right*: Cell surface area represented as mean value ± SEM of *n* = 4 independent experiments (*n* = 3 for EMPA and ANG + EMPA groups). # indicates *P* < 0.01 vs CTRL. β indicates *P* < 0.01 vs ANG

Previously, we demonstrated that ANG increased cell surface area of H9c2 cardiomyoblasts [19]. In our study, H9c2 cardiomyoblasts were treated with ANG, EMPA, or a combination of ANG and EMPA. H9c2 cardiomyoblasts treated with ANG resulted in a significant increase in cell surface area (100% CTRL vs 168.2 ± 11.1% ANG, *P* = 0.008). H9c2 cardiomyoblasts treated with the combination of ANG and EMPA significantly reduced the ANG-induced increase in cell surface area (168.2 ± 11.1% ANG vs 109.5 ± 2.8% ANG + EMPA, *P* = 0.01) (Fig. 1).

### Expression of SGLT-1 and SGLT-2 protein expression in H9c2 cardiomyoblasts. ANG-induced SGLT-1 protein expression was reduced in the presence of EMPA in H9c2 cardiomyoblasts

**Fig. 2:**
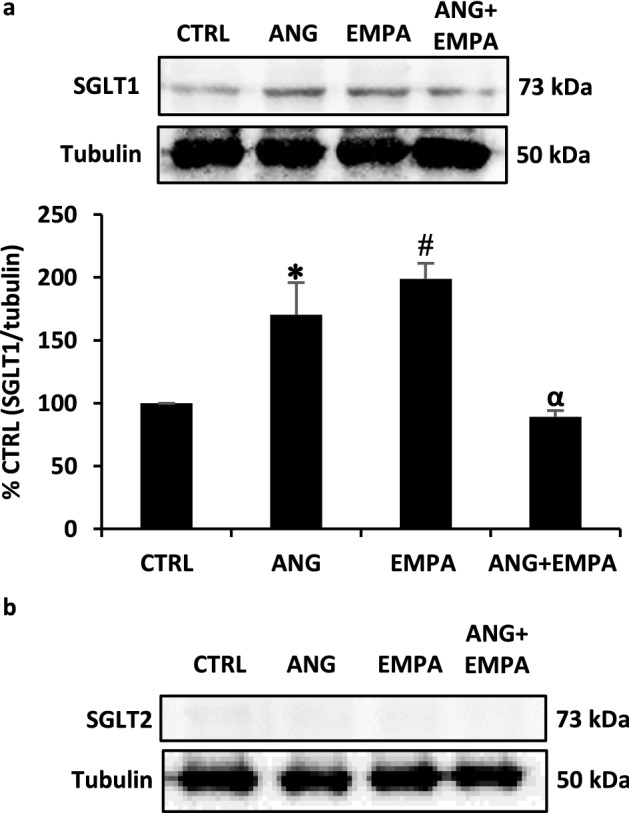
**A** ANG-induced SGLT-1 protein expression was reduced in the presence of EMPA in H9c2 cardiomyoblasts. *Upper*: Representative Western Blot of H9c2 cardiomyoblasts cell lysate probed with anti-SGLT-1. Tubulin was used as loading control. *Lower*: Quantification of SGLT-1 band normalized to tubulin. The bar graph represents the mean value ± SEM of n = 5 independent experiments (*n* = 3 for EMPA, and ANG + EMPA).* indicates *P* < 0.05 vs CTRL. # indicates *P* < 0.01 vs CTRL. α indicates *P* < 0.05 vs ANG. **B** SGLT-2 protein expression of H9c2 cardiomyoblasts with ANG ± EMPA treatment. Representative Western blot of H9c2 cardiomyoblasts cell lysate probed with anti-SGLT-2. Tubulin was used as loading control

H9c2 cardiomyoblasts were treated with ANG, EMPA or a combination of ANG and EMPA. ANG and EMPA independently induced a significant increase in the expression of SGLT-1 protein (100% CTRL vs 170.3 ± 25.4% ANG, *P* = 0.05; 100% CTRL vs 198.8 ± 12.5% EMPA, *P* = 0.01). However, H9c2 cardiomyoblasts treated with the combination of ANG and EMPA significantly reduced the expression of SGLT-1 when compared to treatment with ANG alone (170.3 ± 25.4% ANG vs 89 ± 5% ANG + EMPA, *P* = 0.03) (Fig. 2A). When H9c2 cardiomyoblasts were treated with ANG, EMPA, or a combination of ANG and EMPA, SGLT-2 protein was not expressed in H9c2 cardiomyoblasts as shown in Fig. 2B.

### ANG-induced expression of NHE1 was reduced by EMPA in H9c2 cardiomyoblasts

**Fig. 3:**
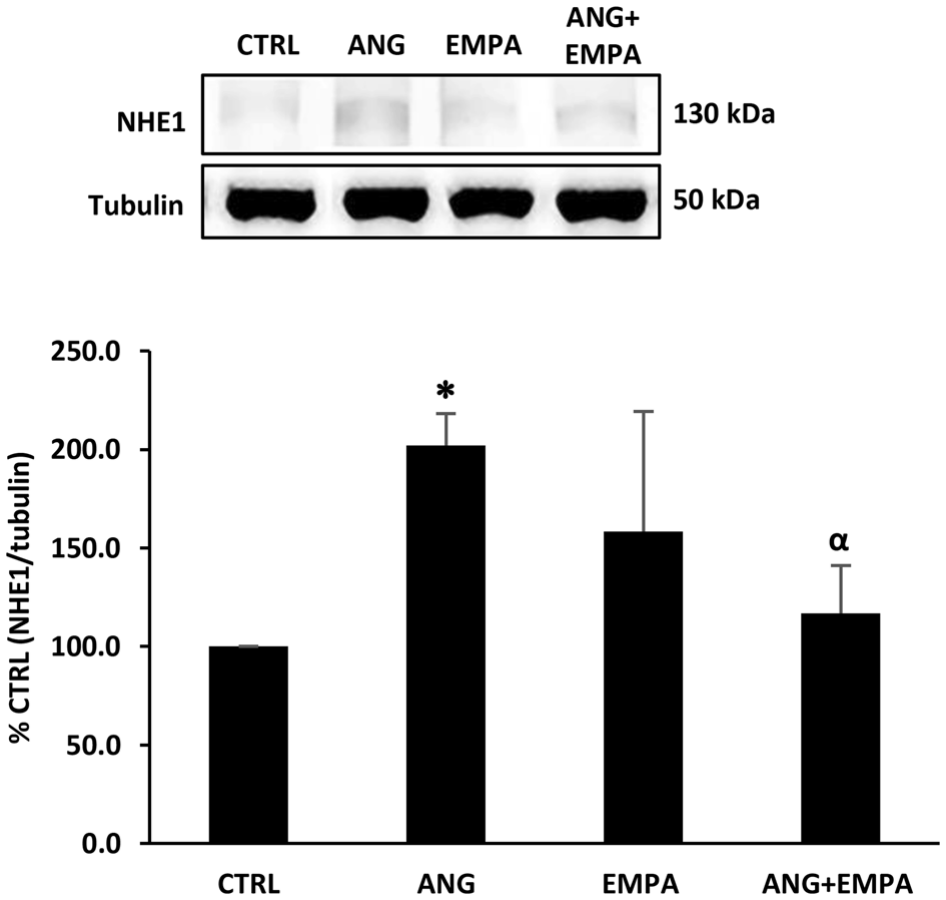
ANG-induced expression of NHE1 was reduced by EMPA in H9c2 cardiomyoblasts. *Upper*: Representative Western blot of H9c2 cardiomyoblasts cell lysate probed with anti-NHE1. Tubulin was used as loading control. Lower: Quantification of NHE1 band normalized to tubulin. The bar graph represents the mean value ± SEM of *n* = 4 independent experiments (*n* = 3 for ANG, and ANG + EMPA). * indicates *P* < 0.05 vs control. α indicates *P* < 0.05 vs ANG

H9c2 cardiomyoblasts were treated with ANG, EMPA, or a combination of ANG and EMPA. ANG treatment significantly increased the expression of NHE1 protein in H9c2 cardiomyoblasts (100% CTRL vs 201.9 ± 16.2% NHE1, *P* = 0.024). This ANG-induced increase in NHE1 protein expression was significantly diminished in the presence of EMPA (201.9 ± 16.2% ANG vs 116.9 ± 24.2% ANG + EMPA, *P* = 0.05) (Fig. 3). Treatment of H9c2 cardiomyoblasts with EMPA resulted in a slight increase in the expression of NHE1 when compared to control; however, this increase was not significant.

### ANG, EMPA, or their combination does not affect the phosphorylation of p90 ribosomal s6 kinase protein expression in H9c2 cardiomyoblasts

**Fig. 4:**
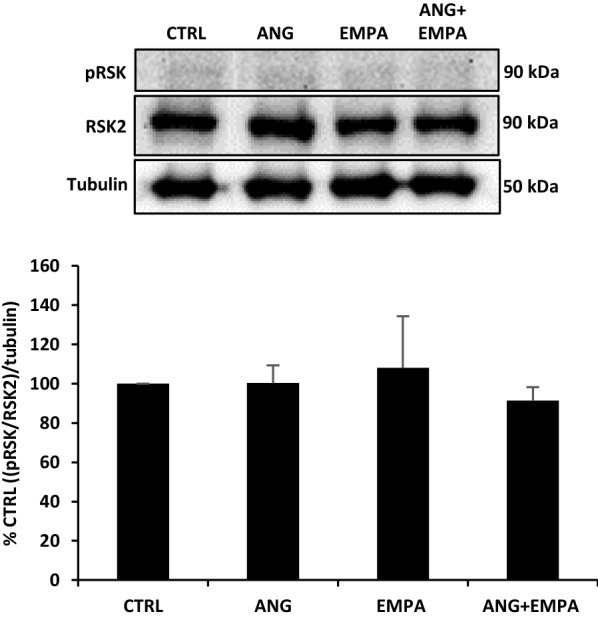
ANG, EMPA, or their combination does not affect the phosphorylation of p90 ribosomal s6 kinase protein expression H9c2 cardiomyoblasts. *Upper*: Representative Western blot of H9c2 cardiomyoblasts cell lysate probed with anti-phospho p90 RSK and RSK2. Tubulin was used as loading control. *Lower*: Quantification of p90 RSK normalized to RSK2 and tubulin. The bar graph represents the mean value ± SEM of *n* = 3 independent experiments

A previous report demonstrated that NHE1-induced cardiac hypertrophy is facilitated through p90 RSK [23]. Additionally, infection of H9c2 cardiomyoblasts with the active form of NHE1 was associated with an increase in the phosphorylation of RSK [9]. In our study, H9c2 cardiomyoblasts treated with ANG, EMPA, or a combination of ANG and EMPA, neither ANG, EMPA nor ANG and EMPA, had any significant change on the expression of phosphorylated RSK in H9c2 cardiomyoblasts (Fig. 4).

### ANG, EMPA, or their combination does not affect the phosphorylation of Akt protein expression in H9c2 cardiomyoblasts

**Fig. 5:**
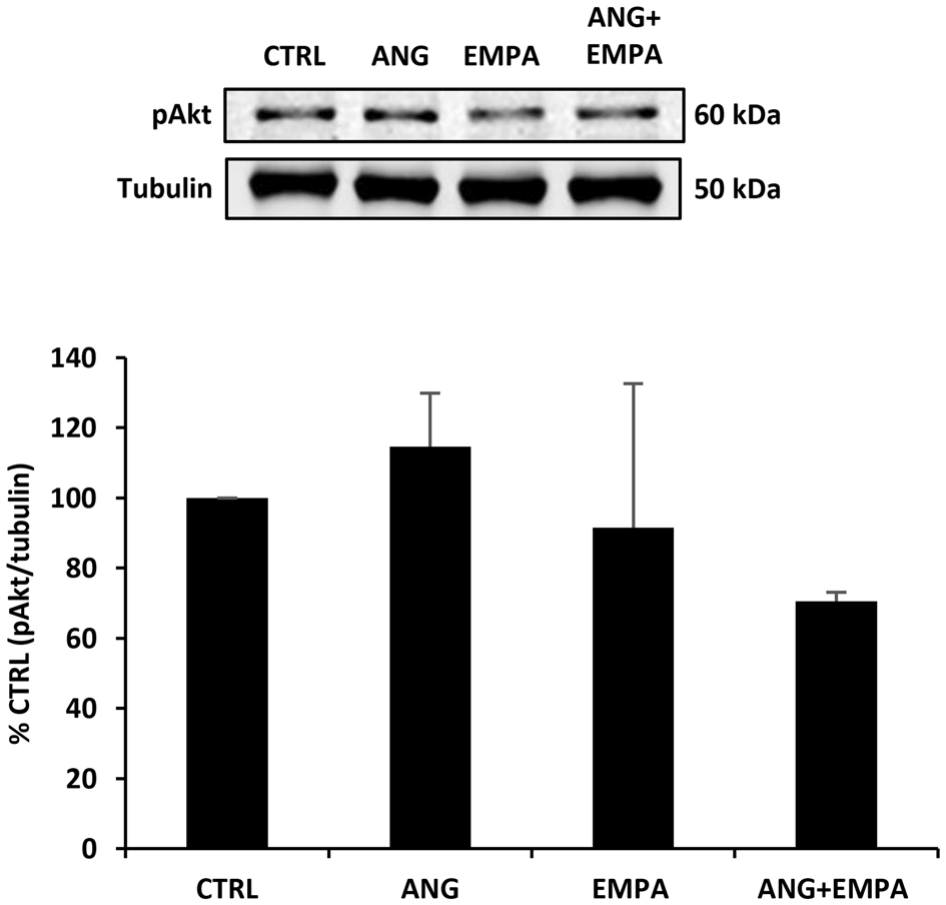
ANG, EMPA, or their combination does not affect the phosphorylation of Akt protein expression H9c2 cardiomyoblasts. *Upper*: Representative Western blot of H9c2 cardiomyoblasts cell lysate probed with anti-phospho-Akt. Tubulin was used as loading control. *Lower*: Quantification of phospho-Akt normalized to tubulin. The bar graph represents the mean value ± SEM of *n* = 4 independent experiments (*n* = 3 for EMPA, and ANG + EMPA)

H9c2 cardiomyoblasts were treated with ANG, EMPA, or a combination of ANG and EMPA. Western blot result showed that neither ANG nor EMPA had any significant change in the expression of phosphorylated Akt in H9c2 cardiomyoblasts (Fig. 5). The combination of ANG and EMPA resulted in a trend toward decrease in the protein expression of phospho Akt; however, this was not significant.

## Discussion

### ANG-induced cell hypertrophy of H9c2 cardiomyoblasts was reduced in the presence of EMPA

Previous studies on H9c2 cardiomyoblasts showed that ANG-induced hypertrophy [17, 18]. This is in accordance with our results, which showed an increase in cell area of H9c2 cardiomyoblasts when treated with ANG. Our results also demonstrated that ANG-induced hypertrophy was significantly diminished in the presence of EMPA. A similar reduction in hypertrophy was demonstrated in a recent study done on cardiac fibroblasts isolated from human atrial tissue; the group of cells treated with EMPA showed a smaller cell size with fewer extensions [24].

### SGLT-1 protein expression in H9c2 cardiomyoblasts was increased following treatment with ANG or EMPA

Our results demonstrated that SGLT-1 protein expression is significantly increased in H9c2 cardiomyoblasts when treated with ANG and EMPA individually. This supports findings from a recent study which demonstrated that changes in the activity of the intrarenal RAAS are positively correlated with changes in endogenous SGLT-2 expression levels and that ANG dose-dependently increases SGLT-2 expression in vitro [25]. Heart failure has been demonstrated to activate both systemic and intrarenal RAAS [25] and human SGLT-1 mRNA expression has been shown to be significantly increased in ischemic cardiomyopathy [26]. Thus, changes in RAAS could contribute to SGLT-1 expression and would suggest the therapeutic use of SGLT-2 inhibitors under conditions of increased RAAS.

### SGLT-2 protein expression was not expressed in H9c2 cardiomyoblasts

Our results demonstrated that H9C2 cardiomyoblasts do not express SGLT-2 protein as shown in Fig. 2. This aligns with recent studies which suggest that SGLT-1 is the isoform expressed in the heart, while SGLT-2 is found predominantly in the kidneys with no expression in cardiomyocytes [27, 28]. Moreover, a recent study demonstrated that SGLT-2 was not expressed in mice hearts [29]. Additionally, a study in rats and mice hearts and human left ventricular biopsy demonstrated that SGLT-1 is expressed in rat, human, and mouse hearts whereas SGLT-2 expression was not observed in any of the cases [30].

### ANG-induced expression of NHE1 was reduced by EMPA in H9c2 cardiomyoblasts

A recent study demonstrated that EMPA decreases myocardial cytoplasmic Na^+^ through inhibition of the cardiac NHE1 in rats and rabbits [12]. Another recent study has demonstrated the inhibition of NHE1 activity in human atrial cardiomyocytes [31]. Moreover, direct inhibition of NHE1 activity by EMPA was demonstrated in ischemic mouse models, which attributed the cardioprotective mechanism to a reduction of autosis [32]. A very recent study has reported that EMPA inhibited inflammation-induced oxidative stress in human endothelial cells via inhibiting NHE and lowering of cytoplasmic Na^+^ level [33]. It is also worth to note that EMPA ameliorated the lipotoxic damage in angiogenic cells potentially through NHE inhibition, which could also support the cardioprotective role of EMPA through inhibiting NHE1 [5].This is in accordance with our results that ANG-induced NHE1 protein expression was significantly diminished in the presence of EMPA. Although SGLT-2 is not expressed in the heart, EMPA can inhibit cardiac NHE1 possibly through a binding site for SGLT-2 on NHE1 [13]. Our findings are not in support of Chung et al. findings which suggest that EMPA does not NHE1; however, in their study, they do not look at NHE1 protein expression [14].

### ANG, EMPA, or their combination does not affect the phosphorylation of p90 RSK and Akt protein expression

p90 RSK is a key NHE1 kinase since p90 RSK phosphorylates NHE1 serine 703 [34] and hence is a vital signaling protein for sustaining regular cardiac function. We have previously reported that NHE1-induced cardiac hypertrophy is facilitated through p90 RSK [23]. In the current study, we did not observe significant changes in p90 RSK protein expression. However, p90 RSK is activated by phosphorylation and it is a quick process and time-dependent process that might be altered during the 24-h incubation period and requires further validation. We have also demonstrated that there was no significant change in Akt protein expression. The absence of evidence for contributions from p90 RSK and Akt to cardiac remodeling may highlight the potential involvement of other proteins. A study performed on female rodent model of diabetes demonstrated that the cardioprotective effect of EMPA is mediated through the serum/glucocorticoid-regulated kinase 1 (SGK1) and the epithelial sodium channel (ENaC) [35].

## Conclusion

Our study showed that ANG-induced hypertrophy of H9c2 cardiomyoblasts is accompanied with increased SGLT-1 and NHE1 protein expression. EMPA reduces ANG-induced cardiac hypertrophy through inhibiting the expression of NHE1. The signaling mechanism involved in the reduced expression of NHE1 by EMPA is yet to be understood.

## Data Availability

All data generated or analyzed during this study are included in this published article.
